# Improving transitions in care for children with complex and medically fragile needs: a mixed methods study

**DOI:** 10.1186/s12887-020-02117-6

**Published:** 2020-05-14

**Authors:** Janet A. Curran, Sydney Breneol, Jocelyn Vine

**Affiliations:** 1 School of Nursing, Dalhousie University, Halifax, Canada; 2 Strengthening Transitions in Care Research Lab, IWK Health Centre, 8th Floor, Children’s Site, 5850/5980 University Avenue, P.O. Box 9700, Halifax, NS B3K 6R8 Canada

**Keywords:** Pediatric, Chronic conditions, Medical complexity, Discharge, Transitions in care, Mixed methods, Patient-oriented research

## Abstract

**Background:**

Children with medical complexity are a small yet resource intensive population in the Canadian health care system. The process for discharging these children from hospital to home is not yet optimal. The overall goal of this project was to develop recommendations to be included in a provincial strategy to support transitions in care for children with complex and medically fragile needs.

**Methods:**

A wide assortment of stakeholders participated in this mixed method, multiphase project. During Phase 1, data was gathered from a range of sources to document families’ experiences transitioning from an inpatient hospital stay back to their home communities. In Phase 2, pediatricians, nurses, and health administrators participated in key stakeholder interviews to identify barriers and facilitators to a successful transition in care for children and families with complex care needs. A multi-sector consensus meeting was held during Phase 3 to discuss study findings and refine key recommendations for inclusion in a provincial strategy.

**Results:**

Six case studies were developed involving children and families discharged home with a variety of complex care needs. Children ranged in age from 15 days to 9 years old. Nine telephone interviews were conducted in Phase 2 with pediatricians, nurses, and administrators from across the province. A variety of inter-institutional communication challenges were described as a major barrier to the transition process. A consistent message across all interviews was the need for improved coordination to facilitate transitions in care. The consensus meeting to review study findings included physicians, nurses, paramedics, senior administrators, and policy analysts from different health and government sectors and resulted in six recommendations for inclusion in a provincial strategy.

**Conclusions:**

This project identified policy and practice gaps that currently exist related to transitions in care for children with complex and medically fragile needs and their families. Our collaborative patient-centred approach to understanding how children and families currently navigate transitions in care provided a foundation for developing recommendations for a provincial wide strategy.

## Background

Children with complex and medically fragile needs are a small yet resource intensive population within the Canadian pediatric health care system [1, 2]. While these children represent less than 1% of the pediatric population, it is estimated they account for one third of pediatric health care spending [2]. Yet, discussion about the needs of children with medical complexity are often overshadowed in national health reform discussions by care of chronically ill adults [3]. Further, estimating the burden of illness on families, communities, and the health care system can be challenging due to the wide variation of health and social needs within this heterogeneous population. Brenner et al. [4] conceptualized “children’s complex care needs [as] the multidimensional health and social care needs in the presence of a recognized medical condition or where there is no unifying diagnosis” [4] (p.1647).

Due to the complex nature of their care needs, these children often experience frequent hospitalizations [1, 2, 5], accounting for approximately 10% of pediatric admissions and 25% of hospital days [6]. In comparison to children without complex chronic conditions, children with medical complexity experience almost 9 times more inpatient visits and 17 times greater inpatient costs [7]. Despite this high inpatient use, it is widely recognized that much of the care these children require could be provided in their home and home communities [8–10]. With as many as 89% of children with complex chronic conditions being discharged from hospitalized settings [11], a well-coordinated and comprehensive transition from hospital to home is essential for improved patient and family outcomes and efficient use of health care resources. However, successful transitions in care for this population are characterized by a number of challenges. As many as 13 physicians from 6 distinct medical specialities and numerous other care team members across the health, educational, and community settings may be involved in the care of these children and families. This sizable care team extending across multiple services and sectors creates the potential for gaps in care coordination and communication [2]. In addition to these factors, their care needs may include dependence on medical technology at discharge (i.e. ventilator, feeding tubes, etc.), requiring adequate support in their home community which can place this vulnerable population at an even greater risk for adverse outcomes or hospital re-admissions, particularly in geographically dispersed communities [2, 8, 12, 13]. There has been increasing discussion in the literature over the past 10 years surrounding pediatric complex care, including new models of service delivery to support children with complex care needs and their families in the community [14, 15]. The American Academy of Pediatrics has advocated for the Medical Home Model, grounded in family-centered primary care services, as a comprehensive, community based model of service for all children with complex care needs and their families [16]. However, current health care structures in North American are not designed to effectively support these principles of care [17].

With the multiple stakeholders involved in the care for this population, providing integrated and coordinated care for children with medical complexity in Canada can prove to be challenging. A recent scoping review of the literature revealed a paucity of programs, interventions, or frameworks designed to support the transition from hospital to home for children with complex needs and their families [18]. Further, findings revealed a lack of patient and family-oriented outcome measures, indicating the need for researchers and policy makers to involve families in the research process and tailor forthcoming programs and policies to the individualized needs of this population [18].

The need for clear guidelines and processes for the provision of high-quality care for children with complex and medically fragile care needs during care transitions is critical. While there are isolated tools and agreements in principle to collaboratively develop short- and long-term care plans for these children and their family, there are currently no generalizable transition processes that systemically guide transitions for this vulnerable population in Nova Scotia, Canada.

### Study aim and objectives

This project aimed to develop recommendations to improve the transition from hospital to home for children with complex and medically fragile needs in Nova Scotia. To achieve this aim, the following research objectives were addressed: (1) describe the experience of patients with complex care needs and their families during the transition from a tertiary care facility to their home community; (2) identify perceived barriers and enablers related to the existing transition process, and (3) identify key components for inclusion in a strategy to enhance the transitioning of children and families with complex and medically fragile needs to their home community.

## Methods

**Fig. 1:**
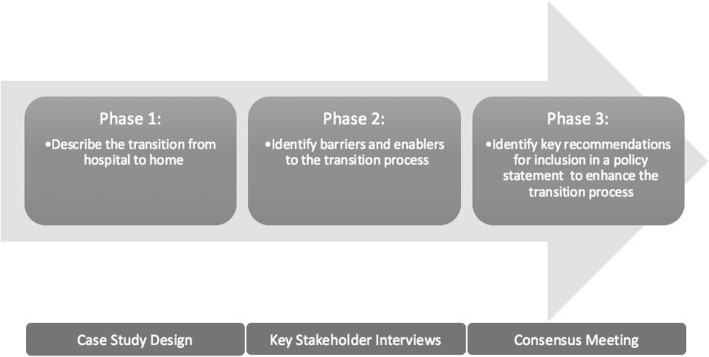
Data collection process

Using an integrated mixed methods approach [19], this project brought together a team of researchers, clinicians, parents/caregivers, and senior-level administrators across the province of Nova Scotia. Data was collected from multiple sources across three phases to achieve a greater breadth and depth of understanding into the existing transition process (Fig. 1) [19].

### Setting

This study took place at a pediatric tertiary care facility located in Nova Scotia, Canada, responsible for the care of children, youth, and women across the Canadian Maritime Provinces. The Canadian Maritimes is composed of three provinces, with a combined population of almost 500,000 children and youth [20]. This health centre has approximately 230 baby and children beds, with estimated 15,000 acute inpatient admissions, 34,000 emergency department visits, and 210,00 outpatient visits annually [21, 22]. Ethical approval was obtained from the IWK Research Ethics Board (Project #1017345). Data collection occurred between 2015 and 2017.

### Phase 1

To address our first objective, a case study design was employed to develop 6 cases representing the experiences of a range of children with complex and medically fragile needs transitioning from hospital to home. We employed maximum variation sampling to ensure a representation of different age groups, levels of complexity, family structures, geography and socioeconomic status levels when possible [19]. Eligibility for this study included parents or guardians of children aged 0–18 years old with one or more chronic conditions that were expected to require specialized care greater than 1 year. This eligibility criteria was created to align with current literature describing children with complex care needs [1, 4, 23] and based on expert opinion from our multidisciplinary research and clinical team. Terminally ill patients were excluded from this study. Clinical leads from each unit in the tertiary care facility invited potential participants according to the eligibility criteria by providing a letter of information describing the study and contact information for the research assistant. The research assistant reviewed the letter of informed consent with potential participants and they were given the opportunity to ask questions before participation.

Each case was informed by multiple sources of data: family, tertiary care provider, and community health care provider interviews and a structured chart audit. Families were asked to identify one primary caregiver to complete the interviews and all data collection measures. All providers involved in the care of these children were invited to participate in our study. Data collection occurred at four points in time: (1) two weeks prior to discharge (T1); (2) one day prior to scheduled discharge (T2); (3) one week following discharge (T3); and four weeks following discharge (T4). Families were asked to complete the SF-36 tool at T1 and T4 to estimate disease burden [24]. The State Trait Anxiety Inventory (STAI) was used to assess parental anxiety and measured at T2 and T3 [25] and the Brief Cope was used to assess parents coping strategies at T2 and T4 [26]. Our approach to data collection attempted to balance understanding a range of patient, family and health system factors relevant to the transition from hospital to home while being mindful of not contributing to caregiver burden during the transition home. The measures were chosen to capture the experiences and potential changes in stress and coping at different time points during the transition from hospital to home. The child’s primary caregiver also participated in individual interviews at three time points: T1, T2, and T3. A semi-structured interview tool, developed by the research team, was used to explore caregiver’s knowledge, attitudes, and beliefs about the discharge process at each time point. Tertiary care and community health care providers who were directly involved in providing care to the patient and family were also interviewed on a single occasion after the child’s discharge from hospital. A semi-structured interview guide was also used to explore provider’s knowledge, attitudes, and beliefs about the discharge process. All interviews were audio-recorded and transcribed verbatim. Interview transcripts were sorted by case and managed in NVivo 11 Qualitative Data Analysis Software [27]. Transcripts were coded by two independent reviewers. A content analysis approach was used to identify important barriers and enablers of the discharge process in each case [28]. Reviewers met to compare findings and coding discrepancies were resolved through discussion and consensus.

### Phase 2

To address our second objective, semi-structured telephone interviews were conducted with key stakeholders across the province to identify barriers and facilitators related to the transition from hospital to home for children with complex and medically fragile needs. An initial list of potential community stakeholder participants was identified through consultation with members of the executive leadership team of the tertiary care centre. Subsequently, we used a snowball strategy inviting study participants to identify additional potential provincial stakeholders. A letter of invitation was sent to potential participants via email. Data was collected through individual telephone interviews and verbal consent was obtained from all participants. A semi-structured interview guide, based on the TDF [29, 30], was used to explore participant’s experiences, attitudes and beliefs about supporting children and families through transitions in care. This interview guide was initially drafted by the principle investigator (JC) and further refined by our team of researchers and clinicians. The interviewer (SB) used prompts when appropriate to encourage participants to elaborate on their experiences and opinions. Interviews were recorded and transcribed verbatim by an experienced transcriptionist. One reviewer (SB) performed deductive content analysis to sort barriers and facilitators arising from each transcript according to the 14 domains of the TDF [29, 30]. Coding began immediately after the first interview and a second reviewer (JC) validated the coding in 40% of the transcripts by independently coding the first and every other transcript. Participant recruitment was deemed complete and data saturation was achieved when no new barriers and facilitators were identified in the transcripts [31]. Subsequently, one reviewer (SB) conducted a thematic analysis to identify important themes related to the barriers and enablers [32]. Frequent checks and collaboration with research team members occurred to discuss themes as they emerged.

### Phase 3

To address our final research objective, we hosted a half-day multi-disciplinary stakeholder meeting to review findings and identify and refine key recommendations for inclusion in a policy statement. Our guiding framework for the meeting was underpinned by an adapted consensus-oriented decision-making process; steps included: (1) framing the topic (through our data); (2) open discussion (of data and analysis); (3) identifying underlying concerns; (4) collaborative strategy building; (5) choosing a direction; (6) synthesizing a final strategy/proposal; and (7) identification of next steps and roles [33].

## Results

### Phase 1

Six cases representing the transition experience for a range of children with complex and medically fragile needs were created. These cases included children with an age range of 15 days to 9 years and were followed by as many as seven different speciality services. Case characteristics are summarized in Table 1. Table 2 provides an overview of the Brief Cope Inventory scores for all cases. A summary of the thematic cross case analysis can be found in Table 3. The following section provides a brief overview of the key features of developed case studies. Geographical distances from the tertiary care centre have been categorized dichotomously to: (1) less than an hour drive and (2) more than an hour drive. A total of 34 health care providers were interviewed across the cases and represented a range of professions which included pediatricians, physiotherapists, social workers, pharmacists, neonatologists, transition coordinators, nurse practitioners, occupational therapists, family care coordinators, dieticians, oncologists, and plastic surgeons.

**Table 1:** Case characteristics with STAI and SF-36 scores

Case #	Child’s age	Child’s diagnoses	Single or dyad household	Support systems	STAI weighted scores^a^ (T2)	STAI weighted scores^a^ (T3)	SF-36 (T1) scores^a^	SF-36 (T4) scores^a^
**Case #1**	2 months	Cardiac Septal Defect, Hip Dysplasia	2	Has family support and a family member with medical training	36	40	PCS^b^: 53.02 MCS^c^: 51.62	PCS: 58.92 MCS: 55.73
**Case #2**	15 days (adjusted age)	Genetic Condition	2	Family live close by in home community	20	21	PCS: 28.99 MCS: 53.61	PCS: 53.27 MCS: 53.15
**Case #3**	10 years	Osteomyelitis, Home IV Antibiotics	2	Family staying in home temporarily to ease the stress of the transition. Family live in close proximity, neighbor is a medical professional.	36	48	PCS: 56.96 MCS: 57.87	PCS: 58.74 MCS: 42.12
**Case #4**	5 months (adjusted age)	Home NJ Feeds	1	Co-parent lives short distance away. Family visting from out-of-province to provide support. Co-parent’s family supportive.	20	20	PCS: 55.04 MCS: 52.40	PCS: 51.46 MCS: 58.00
**Case #5**	1 year	Esophageal Defect, Rehab	2	None identified	46	45	PCS: 61.16 MCS: 42.29	PCS: 61.3 MCS: 45.18
**Case #6**	9 years	Leukemia, Rehab, Ulcerative wounds	2	Family of both caregivers live in close proximity. Family friend who is a retired health care professional	59	48	PCS: 57.95 MCS: 27.98	PCS: 60.22 MCS:45.97

^a^*SF-36* Short Form Health Survey

^b^*PCS* Physical Component Score

^c^*MCS* Mental Component Score

**Table 2:** Case study brief cope inventory scores: T2 and T4

Composite scale	Domain	Case 1 (T2)	Case 1 (T4)	Case 2 (T2)	Case 2 (T4)	Case 3 (T2)	Case 3 (T4)	Case 4 (T2)	Case 4 (T4)	Case 5 (T2)	Case 5 (T4)	Case 6 (T2)	Case 6 (T4)
Dysfunctional strategies	Self Distraction	5	5	5	3	2	3	2	2	3	4	2	4
	Self-Blame	4	2	2	2	2	3	2	2	2	3	2	2
	Denial	3	2	5	4	2	2	2	2	2	2	2	2
	Substance Use	2	2	2	2	2	2	2	2	2	2	2	2
	Behavioural Disengagement	2	2	2	2	2	3	2	2	2	4	2	2
	Venting	4	4	2	1	2	4	2	2	5	5	3	2
Problem-focused	Planning	4	2	7	8	6	4	5	3	8	6	6	7
	Active Coping	3	6	8	8	6	6	4	2	7	5	8	6
	Use of Instrumental Support	5	5	5	5	5	6	6	2	7	4	4	5
Emotional focused	Positive Reframing	8	6	1	5	5	5	2	2	8	7	3	3
	Humour	3	2	2	2	3	2	2	2	5	5	2	2
	Acceptance	8	6	8	4	7	7	5	2	8	7	6	7
	Religion	4	2	2	2	7	7	2	2	2	2	8	8
	Use of Emotional Support	6	8	7	7	7	8	2	4	6	4	4	7
	Total	61	54	58	55	58	62	40	31	67	60	54	59

*Note: Scores range from 2 (Low) up to 8 (High) [26]

**Table 3:** Cross case analysis

Overall case themes	Case study #					
	1	2	3	4	5	6
Trust with HCPs	**√**	**√**	**√**	**√**	**√**	**√**
Discharge process lead by one HCP	**√**				**√**	**√**
School participation			**√**			
Community resources	**√**	**√**		**√**	**√**	
Social support networks		**√**			**√**	
Communication between HCPs			**√**	**√**		
Access to respite care					**√**	
Family socio-emotional coping post-discharge						**√**

#### Case 1

This case examined the hospital to home transition of a 2-month-old infant with a congenital atrial septal defect and hip dysplasia who was discharged home post-operatively for ongoing monitoring and rehabilitation. The family lived more than an hour drive from the tertiary care centre and had experienced multiple admissions. Key features of this case included: (1) family concerns regarding trust with certain members of the health care team and their level of knowledge regarding their child’s condition and (2) extensive collaboration between different care teams which was facilitated by a Nurse Practitioner. Summary measures of physical (PCS) and mental (MCS) health and anxiety are unremarkable (Table 1). When compared with other cases in this study, this family leveraged more dysfunctional versus problem focused strategies across the transition from hospital to home (Table 2).

#### Case 2

This case involved a 15-day-old infant born at 35 weeks gestation with fragility and a genetic anomaly. The family lived within 1 h drive from the pediatric tertiary care centre which meant that certain elements of care could remain the responsibility of the tertiary care centre’s perinatal team. Key features of this case included: (1) a strong sense of support surrounding the caregivers and child from family members and the tertiary and primary care team, which helped ease and support the transition home; and (2) concerns expressed by a member of the tertiary care team regarding inadequate resources in the local community to address nutritional needs of the infant, which led the perinatal team to closely follow feeding and weight gain while at home. Changes were noted in PCS scores from T1 to T4 (28.9 vs 53.7) (Table 1). The family also leveraged a high degree of problem focused strategies during the transition home with a noted increase of positive framing techniques between T2 and T4 (Table 2).

#### Case 3

This case examined the experience of a 10-year-old child with complicated osteomyelitis requiring multiple debridement surgeries prior to discharge to their home community located less than an hour drive from the tertiary care centre. Key features of this case included: (1) a strong sense of trust in the tertiary care team prior to discharge; (2) a decreased level of confidence in the care team post-discharge due to contradictory information received by various providers; (3) communication gaps reported by the family between the home care nurse and the health centre regarding treatment needs; (4) a high level of satisfaction with home nursing services and educational information received regarding their child’s treatment; and (5) an active involvement and cooperation of the child’s school facilitating the accommodation of their medical needs. Estimates of family anxiety and burden were stable (Table 1). Brief cope scores remained relatively consistent between T2 and T4 with a reliance on problem and emotional-focused coping strategies (Table 2).

#### Case 4

This case explored the hospital to home transition of a 5 month-old infant born at 28-weeks gestation with bronchopulmonary dysplasia, gastroesophageal reflux disease and required nasojejunal (NJ) feeding. The more than one-hour drive from the home community to the tertiary care centre presented a challenge, as the there was another other child in home. Key features of this case included: (1) a high level of trust with the care team at the tertiary care centre; (2) difficulty with feeling involved in all care decisions due to the division of time between the health centre and home; (3) concerns from both the care team and the family about the lack of resources to manage the infant’s NJ tube at home; (4) cost associated with formula and prescription; (5) the process of accessing respite care; and (6) limited information provided in discharge summary to adequately inform the primary care physician of the infant’s condition, requiring the caregivers to provide more detailed information about the care plan to their provider. STAI and SF-36 scores were unremarkable (Table 1). When compared with other cases, this family reported limited use of coping strategies at both points in time with problem-focused strategies being most prevalent (Table 2).

#### Case 5

This case examined the hospital to home transition of a 1-year-old infant with gastric atresia with additional complex care needs who had been hospitalized since birth. The family’s home community was located more than 1 h drive from the pediatric health centre. Key features of this case included: (1) a high level of trust in the tertiary care team; (2) other parents on the inpatient unit act as key supports to the family; (3) satisfaction with the care from the local community pediatrician, but dissatisfaction with care received from the community hospital following misplacement of the child’s feeding tube; (4) an advanced practice nurse guiding the discharge process and using a locally developed tool to guide discharge planning that was distributed to the family and home community to promote continuity of care; (5) a high level of engagement from the family helping to facilitate a smooth transition as reported by the health care team; and (6) a lack of pediatric expertise in community physiotherapy. Compared with other cases this family reported the highest PCS across T1 and T4 and reported higher anxiety scores across T2 and T3. (Table 1) This family also reported the highest rate of dysfunctional strategies at T4 when compared with other cases (Table 2). Further, there was a reported decrease in the use of instrumental support once home in the community (Table 2).

#### Case 6

This case examined the transition of a 9-year-old child diagnosed with leukemia and ulcerative wounds who was discharged to a home community over an hour away following a four-month inpatient stay. Key features of this case included: (1) a high level of trust with the tertiary care centre and the development of strong relationships with the nurses and other families on the unit; (2) concerns about inconsistencies regarding access to supports and resources for parents during inpatient stay; (3) high level of satisfaction with home care services, but a desire for more continuity in nursing care; (4) a ‘family care coordinator’ acted as key contact at the pediatric health centre and kept the family informed regarding the care plan; and (5) the family feeling prepared to care for their child’s medical needs at home but not prepared for the socio-emotional challenges and stress of caring for their child within the home. This case reported the highest anxiety at T2 and T3 with an increase in MCS from T2 to T4 (27.9 to 45.9) (Table 1). The reported Brief Cope scores suggest a preference for problem and emotional focused strategies following their discharge home (Table 2).

### Phase 2

Telephone interviews were conducted with nine health care providers from five health regions in Nova Scotia who support children with medical complexity and their families. Interview participants included four pediatric registered nurses, four community health care physicians, and one administrator. Data saturation was determined after the 9th interview as no new themes emerged [31]. Data was organized using the TDF domains and highlighted potential barriers and enablers to the hospital to home transition for children with medical complexity (See Table 4) [29].

**Table 4:** Reported barriers and enablers to the transition from hospital to home

TDF domain	Theme	Example
Knowledge	Clinical Care Guidelines/Policies	“The only thing that I can think may be… I know that the American Academy of Pediatrics have done a bunch of sort of issues or work around having a medical home back when I was in training”
	Unaware of Supports/Resources	“You know, I guess the big thing in our community is knowing what’s out there for them, even from an acute care side, whether it’s the [pediatric tertiary care facility]. If you have a child that’s going home, being discharged from the [pediatric tertiary care facility] you don’t necessarily know all the supports that are in all the home communities that these children are going to. And I think we’re the same way, is that we don’t always realize all the supports that are in place.”
Skills	Psychomotor Medical Skills	“You know, we do chemotherapy here. We look after port-a-caths… Vagal nerve stimulator[s], we know how to use the magnet on it that we’ve trained up on. So our nursing team is very capable of and eager to learn things to be able to keep these kids here and not travelling back and forth as much. We just have to know about it.”
Behavioural regulation	Ideals (Need for Comprehensive Care Coordination)	“I think there needs to be care coordination on both ends. I think there needs to be good discharge planning from the [pediatric tertiary care facility] side. But I also think there needs to be somebody to provide that discharge planning too in the local community. So there needs to be pediatric liaisons in the communities to give that information too.”
Beliefs about capabilities	Health Care Provider Confidence	“But the overwhelming theme there is that we have a system that isn’t tuned to the needs of children and care for children outside of the specialized centre. So you know… And people will say to you very directly, “I don’t have pediatric experience, and I’m not comfortable.”
	Parental Confidence	“I’ve seen it with our newborns that come back that have been born premature and have been in NICU, and now they’re doing well, they’re there just to grow. You know, they don’t need the monitoring, they don’t need that intensive level of care. And we find parents have a very difficult time understanding that the baby is now theirs to take care of. You know, they’re not ready that now they’re on the normal care track that they would have been on had baby been born at term.”
Beliefs about consequences	Family Burdens	“Because you’re worried enough about your child, you don’t need to worry that, you know, is the community ready, are the staff prepared? That, you know, should we land in the emergency room in 2 days’ time, are they going to know who my child is? Are they going to know what he or she needs? Are the staff at the nearest pediatric unit prepared to take care of them or know what to do if something should arise?”
	Hospital Readmissions	“I think the consequences is sometimes it leads to increased admissions into the acute care unit.”
	Breaks in the Continuum of Care	“Because sometimes they might end up presenting back to our emergency department, for example, before they’ve seen any of us….They might end up coming in on a Friday night and seeing one of my other pediatric colleagues that didn’t get any of the documentation I received.
	Preventable Adverse Outcomes	“Well, I think we may miss some opportunities to avoid or to monitor for complications. And so in that way, you know, even if nothing happens, we haven’t been there being as diligent perhaps as we could be for quality assurance and ensuring that the best health care is being provided.”
Goals	High Importance to Improve Transitions in Care	“You know, especially as we move forward and these [kids with complex needs] start growing up … they have more complexities on what they’ve already had with their original diagnosis, it’s something that’s quite important.”
Environmental context and resources	Discharge Communication and Coordinator Processes	“But it would really be knowing even ahead of time if there’s a [child] that is going to come home, what the expected date of discharge would be, what the expected needs might be, what resources, those kind of things.”
	Community Resources	“Well, we’ve started our own program here…. And it only encompasses certain children that we accept into our program. It doesn’t include all children. But those kids that are high needs kids that are in our program, then we follow them more closely. So we know their movement, I guess. So we know when they’ve been at the [pediatric tertiary care facility] and that they’re say coming home, and they have high care needs. But somebody that doesn’t fall into our program, there is nothing in place.”
	Pediatric Tertiary Care Facility Resources	“So one of the things that we use quite frequently with [children with cancer] is the family care coordinator (FCC) model. Or in cancer care in the adult world, they have cancer patient navigators. And so those the FCC, they’re assigned so many oncology patients. And they really kind of run the show. They know the ins and outs. They know what’s going on with them, treatment protocols, all that kind of thing.”

#### Knowledge / skills

The majority of participants were not aware of formal guidelines or policies to help support or inform the transition from hospital to home for children with medical complexity and their families. Guidelines that were discussed included the medical home model from the American Academy of Pediatrics [16], Toronto Sick Kids Complex Care Program [34], and condition-specific health centre policies. A number of health care providers indicated a lack of awareness of the range of resources accessible in the community and the tertiary care facility for both families and health care providers to support this transition in care. Further, given that some smaller communities may go many years in-between assuming care for a child with complex care needs, a lack of experience of how the transition process should occur was reported. Participants also mentioned specific psychomotor skills that may be required by the community health care providers to care for the complex medical needs of these children.

#### Behaviour regulation

Participants spoke of several systems and resources that could be used to support smoother, more effective, and efficient transitions from hospital to home for children with medical complexity and their families. This included: (1) prompt and consistent communication to the appropriate health care providers/services; (2) care coordinators located in the community and the tertiary care centre to oversee the coordination of care and support families; (3) teleconferences between all members of the care team; (4) written care plans provided to both families and providers in advance of discharge from the tertiary care facility; (5) access to additional speciality health care services within the community; and (6) online repository or system to store and organize existing community resources, pediatric tertiary care centre resources, policies, and clinical care guidelines.

#### Beliefs about capabilities

Health care providers highlighted the need for both expertise and confidence in caring for the complex pediatric population to ensure optimal care is provided. Given the rurality of many of these communities, community health care providers may have limited encounters with pediatric patients resulting in a decreased confidence in caring for their unique needs. Practitioners also spoke of the varying parental confidence levels during the shift in care responsibility. Parents must be adequately prepared to transition from a secure hospital environment to their homes where health care providers will not be directly overseeing their child at all times.

#### Beliefs about consequences

Study participants discussed various consequences that may arise without proper structures and processes in place to support the hospital to home transition for children with medical complexity and their families. These included: (1) family burdens (i.e. travelling unnecessarily to and from the tertiary care centre); (2) hospital readmission; (3) interruptions in continuum of care (i.e. providers not having access to the appropriate information); (4) preventable adverse events; and (5) poor reintegration into community (i.e. returning back to school).

#### Goals

All study participants believed the transition from hospital to home for this vulnerable population was of great importance. When asked to rate the importance of improving this transition in care on a scale from 1 to 10, with 1 being not important, and 10 being very important, participants reported an average of 9.3/10 (range 8–10). Participants highlighted the importance of improving this transition in care to ensure the delivery of patient-centred care.

#### Environmental context

A common theme across all interviews was the perceived gap in efficient communication between the tertiary care centre and the receiving community providers. Several community practitioners noted that being consulted early on was a key facilitator to the transition process. Health care providers also discussed the inability to access certain discharge summaries and patient charts and reported not receiving care plan information in a timely manner.

The variability in resources located in the home communities was discussed as both a barrier and enabler to the transition process. Some communities reported having resources such as advance practice nurses, travel funds, and home care services that help support these children and families in the home. However, lack of speciality resources was highlighted as a barrier to providing adequate care for children with complex needs in some rural communities. Participants also discussed various resources located at the pediatric health centre that were helpful in supporting smooth transitions in care. These included care coordinators, perinatal follow-up teams, social workers, clinical nurse specialists, specialty physicians and nurse practitioner. Participants in the community frequently mentioned the importance of a having access to a key worker or coordinator assigned to each child and family.

### Phase 3

Two members of the research team (JC, SB) facilitated a consensus meeting with 18 key stakeholders from across Nova Scotia. Consensus meeting participants included community and tertiary care health care providers, paramedics, senior administrators and policy analysts from the health and education sectors who were not involved in phase 2 data collection. The consensus meeting lasted 2 hours. Key findings from Phase 1 and 2 were presented along with 6 proposed recommendations for inclusion in a provincial policy statement. Recommendations were developed using a behaviour change lens to act as a starting point in addressing the challenges arising from phase 1 and 2 findings. Key priorities in the development of the recommendations included building on existing programs and infrastructures and working with a range of stakeholder groups to co-develop new resources. We also recognized the need to make these resources accessible to end-users across the province, regardless of geographical location. Each recommendation was discussed and revisions were made through a process of consensus as described previously in the methods section. Two points of discussions resulted in modifications to the recommendations. First, consensus meeting participants agreed there was a need for a shared conceptual definition for children with complex and medically fragile needs to orient the present discussion, as well as future clinical and research initiatives examining this population. A number of published definitional frameworks were discussed and participants chose Cohen et al.’s definitional framework as the most suitable given the recent work using this framework in a Canadian context [1, 2]. Second, one of the recommendations included the need for a care coordinator within each provincial health zone to act as a liaison and resource for families and health care providers. Following a lengthy discussion, consensus meeting participants recommended that the coordinator role should be specified as an advanced practice nurse. See Table 5 for a complete list of final recommendations.

**Table 5:** Recommendations for inclusion in provincial policy statement

Adopt the Definitional Framework for Children with Medical Complexity (Cohen et al., 2011) to identify children with intensive care needs in the province of Nova Scotia that are not easily met under existing policies and services.	
Work with existing provincial programs and services (i.e. Continuing Care) to develop policies and tools that are unique to a pediatric population.	
Develop a role for a pediatric advanced practice nurse in each health zone in Nova Scotia to act as a liaison/resource between the tertiary care facility and children discharged with medical complexities, their families and their health care providers to coordinate care and lead capacity building and education initiatives with local health care providers, children, and families.	
Develop a comprehensive discharge plan for every child with complex care needs. The plan must be co-developed and approved by a discharge planner/advanced practice nurse, parent or caregiver-home, or community discharge coordinator prior to discharge from the pediatric tertiary care facility and will consider the medical, psychosocial and developmental requirements for patients to successfully transition back to their home community.	
Develop a Complex Care Information Repository (CCIR) for health care providers, administrators, patients and families to store and organize key resources (contact information for key personnel, clinical practice guidelines, community/hospital resources, etc).	
Develop an Educational Outreach Strategy to address the knowledge, skills and competency needs of health care providers across Nova Scotia who care for children with medical complexity.	

## Discussion

This project aimed to develop recommendations to improve the hospital to home transition for children with complex and medically fragile needs and their families in one Canadian province. This multi-phased research initiative incorporated the perspectives and experiences from families, administrators, and health care professionals to co-develop 6 key recommendations for policy and practice change. In addition to these recommendations, examination of study findings revealed a variety of gaps and areas for future research to optimally support these families during transitions from hospital to home.

Understanding the experiences and perspectives of families of children with medical complexity is essential for designing policies and systems to improve transitions in care for this vulnerable populations [35]. Cases included in this study represented a range of experiences and complexity. Across all cases, there were a number of similarities between family experiences transitioning from hospital to home. Prior to discharge, families reported feeling prepared to manage their child’s medical care, and eager to return home once their child was medically stable. Nurses were identified as a key member of a child’s care team within the inpatient setting. They acted as both an educational resource, care coordinator, and emotional support, facilitating a sense of empowerment and security within families. This finding is consistent with the literature describing the role of nurses in supporting seamless discharge from hospital to home [36, 37]. Similar to other studies, parents in this case sample also identified other parents as an important source of support [38].

Divergence appeared across cases in regard to the availability of resources and support after returning home from hospital. While a number of parents reported having access to appropriate resources in their community, some families reported feeling inadequately supported to assume the various medical, social, and emotional care needs of their child in addition to their daily responsibilities (e.g. work, family, personal needs). Parent readiness for caregiving at discharge can shape the families ongoing experience [8]. Ideally, the shift in responsibility for care from hospital to home should be negotiated with parents prior to discharge [39]. The health care system is beginning to address the shift in care of these children from hospitalized to community-based settings, but work still remains to be completed to optimally support these children and families to flourish in the home [15]. There have been a number of emerging models of care to support advanced health care in the community. These proposed models range greatly in regards to their characteristics, including being both hospital- and primary care- managed, further highlighting the lack of agreement surrounding the optimal model of care for these children [14, 16]. It is additionally important to recognize that caring for the health of the family as a whole is critical, as the child’s health is often linked with that of their caregiver [40, 41]. In designing an optimal health care system for individuals with complex care needs, the Commonwealth Fund released 10 recommendations for policymakers [27]. Of these recommendations, one clearly outlines the need for greater support for caregivers, suggesting that “support might include respite care to provide relief for caregivers and assistance to help them look after their own health” ([27], p.4). The crucial need to enact this recommendation is further confirmed by families of children with complex care needs reporting higher levels of depression [42, 43], stress [44], poorer general health [43] and feelings of isolation [45]. One scoping review conducted by Edelstein, Schippke, Sheffe, and Kingsnorth (2016) identified a range of interventions aimed to mitigate the stress experienced by caregivers of children with medical complexity living in the community [46]. These programs included care coordinator models, respite care, telemedicine, financial benefits and peer support programs [46]. With the promising results emerging from this review, future high-quality research evaluating the effectiveness of these interventions on caregivers is warranted to improve the support provided to families of children with complex care needs [46].

Attending to the complex multidimensional health and social care needs of this population of children requires a multidisciplinary approach [4]. Timely and effective communication between all members of the care team was identified as an essential component to a successful transition home. Health care providers often reported turning to specific nurses when unsure about details of a certain care plan, with most all providers expressing the desire for a key coordinator assigned to each child with complex care needs and their family to facilitate effective communication. The role of this care coordinator, or otherwise referred to as a ‘key worker’, in the care transition process has been gaining increasing attention over the past number of years [18, 41, 47, 48]. Most recently, the Canadian Association of Pediatric Health Centres has recommended the development of a key worker role to facilitate transition planning and management for all children with medical complexity and their families [48]. Further, the need of a care coordinator role is also mentioned in the Commonwealth Fund’s 10 recommendations for optimal health care for individuals with complex care needs to reduce the risk of fragmented health care delivery and facilitate timely communication amongst the care team [41]. To support this transition, key workers act as a single point of entry, enable team collaboration and streamline essential communication amongst families and health care providers [47, 48]. This key worker is a proposed strategy to mitigate the care coordination and communication challenges associated with what is often a large health team across multiple services and sectors managing the care of these children and their families. Key workers not only streamline team communication, but also play a pivotal role in empowering and engaging families in the child’s care [49]. As the development of this role continues, it is critical that patient-oriented research is conducted to evaluate its effectiveness, make appropriate changes to the roles based on specific contexts and to ensure optimal care delivery for this vulnerable population. An economic evaluation would also be important to estimate the relative costs and consequences of implementing this new role.

Health care providers reported a paucity of knowledge of specific formal guidelines or policies to guide transitions from hospital to home for children with complex care needs. However, informal processes within each discipline were followed and many teams had developed their own discharge planning tools to ensure all appropriate actions and processes were taken to ensure smooth transitions in care. While there are a number of recommendations and guidelines aimed at the supporting the care management of this population [8, 48, 50, 51], there remains a limited number of empirically evaluated programs and interventions informing the transition from hospital to home [18]. Although the transition process should be customized to the individualized needs of children and families, overarching guidelines and structures should to be in place to ensure timely transition planning and continuity in care. However, to ensure the development of relevant, comprehensive, and sustainable policy recommendations, engaging a variety of key stakeholder is crucial [52]. Leveraging research techniques to bring family experiences and perspectives to the forefront of decision making is essential to ensuring family-oriented practice and policy changes are pursued. Further, adopting an integrated knowledge translation approach by partnering with knowledge users throughout the research process can help ensure the knowledge being co-created will be relevant to their needs, and therefore more likely to be adopted into practice and policy [53, 54]. The recommendations stemming from this study were developed by integrating the experiential knowledge from families with the perspectives of stakeholders across the tertiary care, community care, and government sectors. Results from this research serves as a critical foundation for future intervention and program design to support the entire care team, including health care providers, administrators, and families, during the often complex, dynamic, and unique transition from the hospitalized setting back into a child’s home and home community.

### Limitations

There are several limitations to note in this study. Although a range of children, families, and health care providers participated in this study and we leveraged a range of data collection sources and strategies, these findings may not be generalizable to the transition experiences of all children and families with complex care needs. We developed a total of 6 cases to explore a range of experiences transitioning from hospital to home, however we recognize that this is a heterogeneous population of children and our cases may not represent the experiences of all children and families with complex care needs. Further, a comprehensive understanding of the impact of the hospital to home transitions may not have been fully captured within the 1 month follow-up time in the case studies [13]. Only individuals able to speak English were eligible to participate. Children and families speaking other languages may have different experiences transitioning from hospital to home that were not captured by this study.

## Conclusion

Children with medical complexity represent a small but resource intensive portion of the pediatric population in the Canadian health care system. Transitions from tertiary care to home can pose many challenges for children, families and health care providers in the recipient communities. Our project identified policy and practice gaps that currently exist in one Canadian province related to transitions from a tertiary care facility to home communities for children with medically fragile needs and families. Our collaborative patient-centred approach to understanding how children, families, and health care providers currently navigate transitions in care provided a foundation for developing recommendations for a provincial wide strategy.

## Data Availability

The datasets generated and/or analyzed during the current study are not publicly available due issues with privacy and confidentiality but are available from the corresponding author on reasonable request.

## Appendix

**Table 6:** Theoretical Domains Framework Definitions

TDF domain	Definition
Knowledge	“An awareness of the existence of something” (p.88)
Skills	“An ability or proficiency acquire through practice” (p.88)
Memory, attention and decision processes	“The ability to retain information, focus selectively on aspects of the environment and choose between two or more alternatives” (p.88)
Behavioural regulation	“Anything aimed at managing or changing objectively observed or measured actions” (p.88)
Social/professional role and identity	“A coherent set of behaviours and displayed personal qualities of an individual in a social or work setting” (p.89)
Beliefs about capabilities	“Acceptance of the truth, reality, or validity about an ability, talent, or facility that a person can put to constructive use” (p.89)
Optimism	“The confidence that things will happen for the best or that desired goals will be attained” (p.89)
Beliefs about consequences	“Acceptance of the truth, reality, or validity about outcomes of a behaviour or a resolve to act in a certain way” (p.89)
Intentions	“A conscious decision to perform a behaviour or a resolve to act in a certain way” (p.89)
Goals	“Mental representations of outcomes or end states that an individual wants to achieve” (p.89)
Reinforcement	“Increasing the probability of a response by arranging a dependent relationship, or contingency, between the response and a given stimulus” (p.89)
Emotion	“A complex reaction pattern, involving experiential, behavioural, and physiological elements, by which the individual attempts to deal with a personally significant matter or event” (p.89)
Environmental context and resources	“Any circumstance of a person’s situation or environment that discourages or encourages the development of skills and abilities, independent, social competence, and adaptive behaviour” (p.90)
Social influences	“Those interpersonal processes that can cause individual to change their thoughts, feelings, or behaviours” (p.90)

[29, 55]

## Abbreviations

TDF: Theoretical Domains Framework
SF-36: Short Form Health Survey
PCS: Physical Component Score
MCS: Mental Component Score
